# Thank you to our reviewers

**DOI:** 10.1017/cts.2022.14

**Published:** 2022-03-11

We are indebted to the expert referees who have volunteered their time to review submissions for the *Journal of Clinical and Translational Science* in 2021. The editors, authors, and readers are immensely grateful for their thoughtfulness and expertise that have served our journal well.

Maryam Mohamed Abdelkarim

Zainab Abedin

Noura Abul-husn

Erin Abu-Rish Blakeney

Edna Acosta-Pérez

Joan Adamo

Sergio Aguilar-Gaxiola

Monica L. Albertie

Leah Alexander

Ayham Alkhachroum

Emily Anderson

Nicholas Anderson

Bo Angelin

Eric A. Apaydin

Joyce Balls-Berry

Lauren Balmert

Shweta Bansal

Gerhard Bauer

Sarah Beachy

L. Michelle Bennett

Gordon R. Bernard

Laura Mari Beskow

Adil E. Bharucha

Pamela Bhatti

Jason T. Blackard

Irene Blanco

Peter Blankestijn

Liz Bloss

Henry Michael Blumberg

Elaine A. Borawski

L. Ebony Boulware

Barrett Bowling

Donita Brady

Allan R. Brasier

Ann Brearley

Guy Brock

Dana Burshell

John B. Buse

Heather M. Bush

Joan Butler

Alison Caballero

Gina Nicole Calco

Karen Calhoun

Kenzie A. Cameron

Maribel Campos

Felicia Caples

Anne Cappola

Molly Carnes

Karen K. Carter

Lori Carter-Edwards

Eida Castro

David Center

David Chambers

Esther Chang

Alex Cheng

Shimul Chowdhury

Nichelle L. Cobb

Barry Coller

Maricarmen Colon

Michael Conway

Dan Michael Cooper

Linda Cottler

Jennifer Croker

Stephen Crowley

Paula Croxson

John Cullen

Andrew Dahlem

Denise Daudelin

Gaurav Dave

Karina Davidson

James Davis

Mike DeBaun

Willard Dere

Gayathri Devi

Deborah DiazGranados

Mark Dignan

Aileen Dinkjian

Aalap Doshi

Ann M. Dozier

Steven Dubinett

Ron Duran

Nicholas Durr

Carrie Dykes

Milton Mickey Eder

Terri L. Edwards

Mark Effron

Ronit Elk

Vicki L. Ellingrod

Felicity T. Enders

John F. Ennever

Estela Estape

Julio Facelli

Layla Fattah

Jessica M. Faupel-Badger

David Feola

Steven Fiore

Gary S. Firestein

Kevin Fiscella

Garret Fitzgerald

Elizabeth Flood-Grady

Patrick Flume

Mona N. Fouad

Deborah M. Fournier

Floyd Fowler

Caroline Freitag

John A. Gallis

Sunanda Gaur

Saeid Ghavami

Kristine Glauber

Kelly Gleason

Sarah L. Goff

Mitzi Gonzales

Ramkiran Gouripeddi

Tom Greene

Kevin Grumbach

Angela Haczku

Perry Halushka

Brett Harnett

Paul A. Harris

Kevin Harter

Karen Hartman

Sarah Hawley

Tara Helmer

Stuart Henderson

Andi Hess

Katelyn Holliday

John Horigan

Grant Huang

Yuan Huang

Shawna V. Hudson

Joe Hunt

Rebecca D. Jackson

Laura P. James

Dushyantha Jayaweera

Carolynn Thomas Jones

Cathleen Kane

Nick Kenyon

Lisa M. Klesges

Jacqueline Knapke

Ann R. Knebel

Andrew Knighton

H. Robert Kolb

Michael Konstan

Karen L. Kopicko

Rhonda G. Kost

Crystal Krause

Jack Kues

Bethany M. Kwan

Beth LaPensee

Bernard LaSalle

Elyse Lasser

Jerold Last

Bethany Laursen

Colleen E. Lawrence

MinJae Lee

Aaron L. Leppin

Christopher Lindsell

Mike Linke

On-Yee (Amy) Lo

Janet Long

Gaetano Romano Lotrecchiano

Tammy L. Loucks

Vashisht Madabhushi

Eric Mah

Jane Mahoney

Tabassum Majid

James Marcin

Eve L. Marion

Doreen E. Martinez

Karen G. Martinez

Kate Marusina

Tanya Mathew

Wayne T. McCormack

Richard McGee

Aileen McGinn

Kate McGlone West

Julie P. McKeel

Linda McManus

Amanda McMillan

Emma Anne Meagher

Valentina Medici

Paul Meissner

Robin Mermelstein

Allison Metz

Frederick J. Meyers

Lloyd Michener

Marsha Michie

Sean Mooney

Cynthia Morris

Jessica Mozersky

Timothy F. Murphy

Leslie A. Musshafen

Marjorie Neidecker

Jason J. Nichols

Daniel Nishijima

Marie K. Norman

Keith C. Norris

George T. OʼConnor

Robert A. Oster

Drew Paine

Angela L. Palmer-Wackerly

John Parant

Richard Parish

Susan R. Passmore

Cecilia Patino-Sutton

Thomas Patterson

Margaret Paul

Thomas Pearson

Clara M. Pelfrey

Kristina Petersen

Tricia Piechowski

Michael Pilinger

Rubin Pillay

Roy Poblete

Jordan Poll

Leah Pope

Jane Powers

Jill M. Pulley

Susan Pusek

Andrew Quanbeck

Julie Rainwater

Rose Marie Ramos

Damayanthi (Dayan) Ranwala

David Redden

Heather Reisinger

Rachel Richesson

Marisa Rinkus

Marylyn D. Ritchie

Alyssa Robillard

Shari Rogal

Betsy Rolland

Mario Rotondi

Doris Rubio

Eduardo Salas

Maritza Salazar Campo

Elias Samuels

Lauren Sankary

Fernando Santana

Margaret Schneider

Alexandra Scholze

Andrea Scott

Gabriel Shaibi

Prabhu Shankar

Jackilen Shannon

Paula Smailes

Andrew Smith

William Smoyer

Patricia Soranno

Christine Sorkness

Venkatesan Srinivasan

Kimber Stanhope

Louisa Stark

Justin Starren

Scott Steele

Jane Steinberg

Elizabeth Stevens

Kathleen R. Stevens

John F. Stevenson

Daniel Stokols

Kimberly Summers

Mark Supiano

Kathy Sward

Mahanaz Syed

Teresa Taft

Laneshia Tague

Holly Taylor

Nathan Tesch

Beth B. Tigges

Hendry Ton

Robert Toto

Jason Umans

Joseph M. Unger

Apurva Uniyal

Randy Urban

Melissa Valerio

Cheryl Vaughan

Roger D. Vaughan

Joao Ricardo Vissoci

Alfred Vitale

Boris Volkov

Yianna Vovides

Kavishwar Wagholikar

Russ Waitman

Kyle Walsh

Molly Wasko

Karriem Watson

Karen Weavers

Fern Webb

Anne Marie Weber-Main

Kevin Weinfurt

Lisa C. Welch

Leah J. Welty

Karen Wilson

Anthony Windebank

Robyn Woodward-Kron

Kevin Wooten

Juli Wu

Theodore Wun

Yaomin Xu

Joel Yager

Fei Yu

Adrienne Zell

Nanhua Zhang

